# Wireless Displacement Sensing Enabled by Metamaterial Probes for Remote Structural Health Monitoring

**DOI:** 10.3390/s140101691

**Published:** 2014-01-17

**Authors:** Burak Ozbey, Emre Unal, Hatice Ertugrul, Ozgur Kurc, Christian M. Puttlitz, Vakur B. Erturk, Ayhan Altintas, Hilmi Volkan Demir

**Affiliations:** 1 Department of Electrical and Electronics Engineering, Department of Physics, UNAM—Institute of Materials Science and Nanotechnology, Bilkent University, Ankara TR-06800, Turkey; E-Mails: unale@bilkent.edu.tr (E.U.); hatice@ee.bilkent.edu.tr (H.E.); vakur@ee.bilkent.edu.tr (V.B.E.); altintas@ee.bilkent.edu.tr (A.A.); 2 Department of Civil Engineering, Middle East Technical University, Ankara TR-06800, Turkey; E-Mail: kurc@metu.edu.tr; 3 Department of Mechanical Engineering, School of Biomedical Engineering, Department of Clinical Sciences, Colorado State University, Ft Collins, CO 80523, USA; E-Mail: puttlitz@engr.colostate.edu; 4 School of Electrical and Electronic Engineering, School of Physical and Mathematical Sciences, Nanyang Technological University, Singapore 639798, Singapore

**Keywords:** displacement sensor, metamaterial, structural health monitoring

## Abstract

We propose and demonstrate a wireless, passive, metamaterial-based sensor that allows for remotely monitoring submicron displacements over millimeter ranges. The sensor comprises a probe made of multiple nested split ring resonators (NSRRs) in a double-comb architecture coupled to an external antenna in its near-field. In operation, the sensor detects displacement of a structure onto which the NSRR probe is attached by telemetrically tracking the shift in its local frequency peaks. Owing to the NSRR's near-field excitation response, which is highly sensitive to the displaced comb-teeth over a wide separation, the wireless sensing system exhibits a relatively high resolution (<1 μm) and a large dynamic range (over 7 mm), along with high levels of linearity (*R*^2^ > 0.99 over 5 mm) and sensitivity (>12.7 MHz/mm in the 1–3 mm range). The sensor is also shown to be working in the linear region in a scenario where it is attached to a standard structural reinforcing bar. Because of its wireless and passive nature, together with its low cost, the proposed system enabled by the metamaterial probes holds a great promise for applications in remote structural health monitoring.

## 1. Introduction

Structural health monitoring (SHM) is essential to ensuring the reliability of structures and protection of human life using the structures. One of the most important damage indices in SHM is the relative displacement experienced in structural components, including reinforcing bars (rebars) embedded into concrete. The displacement, either in the form of contraction or elongation, can occur due to a sudden impact like an earthquake or can slowly develop over time. The ratio of this relative displacement to the original length of the structure allows for calculation of material strain, and the ratio of the stress to the strain provides the Young's (elastic) modulus, which is an intrinsic property of the structure material. Therefore, measurement of relative displacement provides critical information with regard to the force present on the reinforcing bars, which can be utilized to compute the axial force and moment of a reinforced concrete component. A large number of displacement and strain measurement technologies have been reported in the literature, and it has been identified that sensors that leverage remote measurement via wireless monitoring are of higher importance [1–7]. This is due to their capability to deliver temporal information about structural elements in a completely nondestructive manner. Additionally, using a passive sensor in such a wireless system further avoids the need for an electrical power source connected to the sensor. Previous works that incorporate passive wireless sensors for the purpose of SHM are summarized by Deivasigamani *et al.* in [8].

Metamaterials have increasingly been exploited in numerous applications (negative refraction optics [9], superlenses [10], cloaking [11], *etc*.). Recently, metamaterial-inspired architectures have also been investigated for sensing. Metamaterials can offer higher sensitivity and resolution compared to traditional structures and possess a greater potential to deliver strong enhancement and localization of fields [12]. It has previously been reported that metamaterials can be used in biosensors [13,14], thin-film sensors [15], and wireless strain sensors [16] that operate in the microwave and terahertz ranges. A recent review by Chen *et al.* describes in detail the employment of metamaterials in sensing applications [12]. Among these previous efforts, metamaterials have also been devised by our group for wireless strain measurement in various applications including the evaluation of bone fracture healing and the development of smart bioimplants [17,18].

In this work, a class of wireless displacement sensing system with a metamaterial-based probe integrated on a structural component is proposed and demonstrated. As a unique feature, different than a typical strain sensor, this displacement sensor architecture does not require the strain to be propagated from the surface of interest to the sensor chip. The sensing probe is composed of two moving parts with an electrical connection between these parts. Each part contains a partitioned half of the metamaterial resonator developed for telemetric sensing. These two independent parts are separately fixed on the structural component through point attachments, one point for each part, intentionally avoiding the need for strain propagation to the chip. This is critically important because it eliminates the problem of sensor detachment from the surface, a fundamental problem observed at high strain levels with traditional strain gauges. In the case that the starting distance between these two attachment points are known, it is also possible to calculate the material strain using the displacement information if required.

## 2. Architecture of the Sensor

**Figure 1.**
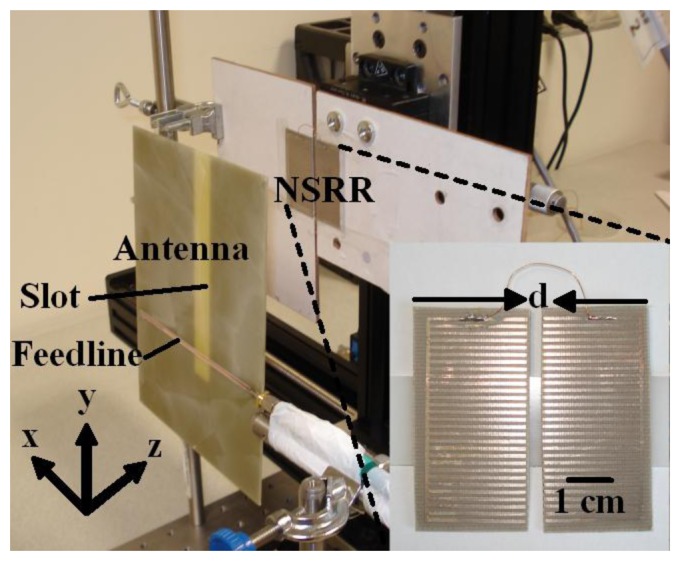
Comb-like NSRR probe and external antenna shown on the test setup, with the comb-like NSRR zoomed in the bottom right corner (inset).

The metamaterial architecture of the sensing probe is composed of a nested split ring resonator (NSRR). The NSRR probe is attached onto the structure whose displacement is to be measured. In addition to the NSRR probe, the proposed displacement sensing system also includes an antenna external to the structure under test, which is used both to transmit signals to and receive information from the NSRR probe (see Figure 1). The NSRR probe is coupled to the antenna within its near field, and displacement on the NSRR is directly observed on the coupled system response. Hence, far-field approximations do not apply, and, as a whole, these two elements constitute a near-field sensing system. The NSRR probe is in comb-like configuration, first proposed by our group in [19]. Essentially, it is a special class of distributed LC resonator whose effective capacitance can be controlled by the number of the comb teeth adapted in the resonator. Owing to the NSRR's near-field excitation response, which is highly sensitive to the displaced comb-teeth over a wide separation, the resulting wireless sensing system exhibits a relatively high resolution (<1 μm) as well as a large dynamic range (over 7 mm), along with high levels of linearity (>0.99 across 5 mm) and sensitivity (>12.7 MHz/mm in the 1–3 mm range).

The comb-like NSRR is symmetrically split into two partitions with respect to the gap between the teeth. These two parts, free to move with respect to each other, are further short-circuited on one end by soldering a thin, loose enough jumper wire to the normally continuous outermost pair of teeth (see Figure 1 inset). This way, when a displacement occurs on the structure on which the NSRR probe is fixed, these two moving parts are separated from (or brought closer to) each other by the amount of the displacement that has formed. This translates into a capacitive change in the NSRR probe, which in turn shifts the spectrum of the coupled system response. Here, for a proof-of-concept demonstration, our comb-like NSRR design incorporates 27 tooth pairs (each pair forming a slit), apart from the normally continuous outermost tooth pair. The number of pairs is optimized in order that the NSRR probe shows a resonance at the operation frequency (approximately 400 MHz). The NSRR was fabricated over a footprint of 47 × 47 mm on a single-sided Rogers Duroid substrate with a relative dielectric constant of 3.2 and a thickness of 0.508 mm. The width of metal lines was fixed at 0.8 mm, and the length of each tooth was set to be 21.6 mm with a gap (split) of 0.8 mm between every pair of teeth.

Previous work by our group used the sensor devices without physically splitting them up and the strain that formed on the structure being measured was propagated onto the sensor chip. This required the sensor to stretch (or contract), thus the sensing was limited to the Young's modulus of the sensor material. The NSRR is made up of mostly a dielectric and it sometimes comprises a ground plane (to capacitively load the meta-structure and further lower the operating frequency if needed), which means that it is not a relatively compliant material. In effect, this constrains the sensor to measuring very small strains (10^−2^ or less) in the linear elastic region of most metals when not partitioned into free moving parts. However, since the current displacement sensor architecture relies on a split facing-double-comb NSRR structure, which allows it to be utilized in two electrically shorted parts, the maximum displacement that can be measured only depends on the jumper length. Therefore, the maximum measureable strain is substantially increased. Here it is worth noting that, while the length of the jumper is typically a few centimeters, it should not be too long since this increases the overall inductance and causes the system resonance frequency to drop out of the antenna bandwidth.

The antenna used in the setup (depicted in Figure 1) is a single-slot microstrip antenna based on the design given in [20] and is modified here to operate around 400 MHz with an approximate bandwidth of 10% where |S_11_| < −10 dB. The slot antenna is excited through a microstrip line along the x-direction at the back side of the substrate, resulting in an x-polarized E-field being transmitted from the slot introduced at the other side of the substrate, which is face to face with the NSRR, and illuminating the NSRR. The splits of the NSRR probe are also x-directed; hence, a strong coupling is formed between the antenna and the NSRR. Here, the usage of an NSRR as the probe enables a much reduced size (4.7 × 4.7 cm) compared to the operating wavelength. This constitutes a distinct advantage since a more compact probe implies that it can be utilized on smaller and thinner structural parts.

## 3. Theoretical Discussion

**Figure 2.**
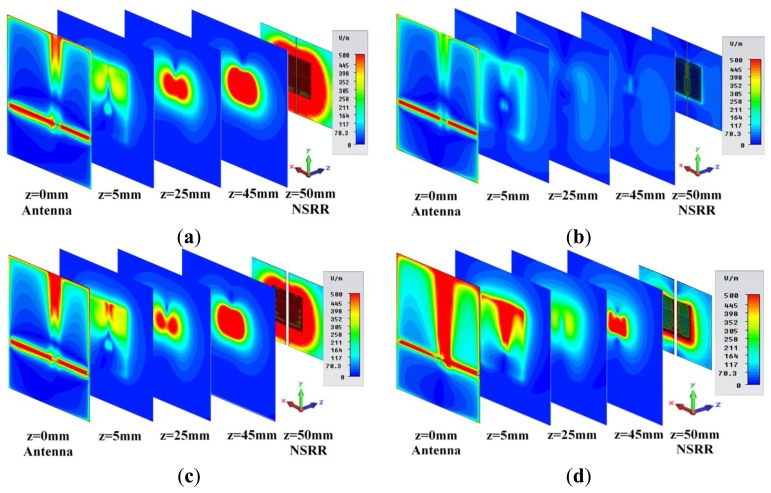
(**a**) Electric field map numerically calculated on the antenna on the 1-mm-separated NSRR probe, and at several cross-sections between the antenna and the probe when the simulation frequency is the resonance frequency of *d* = 1 mm (406 MHz); and (**b**) when *d* = 1 mm again but the simulation frequency is 448 MHz (off-resonance case for *d* = 1 mm); (**c**) Electric field map on the antenna, on the 5-mm-separated NSRR probe and at several cross-sections between the antenna and the probe when the simulation frequency is the resonance frequency of *d* = 5 mm (448 MHz); and (**d**) when *d* = 5 mm again but the simulation frequency is 406 MHz (off-resonance case for *d* = 5 mm).

The fact that the NSRR probe operates in the near field of the antenna provides important advantages in terms of sensitivity. To predict this behavior and develop a better insight for its operation, numerical studies were systematically carried out using the transient solver of the commercial CAD software CST Microwave Studio^®^. These simulations were used to compare the localization of fields on the NSRR when the NSRR is excited within the near field of the antenna for four different scenarios, where the monitoring distance (*D_m_*, which is the separating distance between the antenna and the NSRR) is 5 cm for all cases. The cross-sections of the electric field intensities for these four cases are shown in Figure 2. In the first scenario (see Figure 2a), the gap between the two sides of the comb-like NSRR is 1 mm and the simulation frequency is the resonance frequency corresponding to the 1 mm displacement, that is 406 MHz. As can be observed from the figure, a high localization of fields is achieved on the NSRR for this resonance case. The NSRR creates a local characteristic field (significantly dominant at *z* = 45 mm) which reflects back to the antenna on which we observe the coupled system response. On the other hand, in the scenario presented in Figure 2b, we still have a 1 mm gap between the two parts of the NSRR, but the simulation frequency is 448 MHz which is the resonance case for a 5 mm gap and we can observe that the localization on the NSRR is much poorer and the field that is reflected back to the antenna is much less. This is also the case that is demonstrated in Figure 2c,d, where the gap between two NSRR parts is 5 mm and the simulation frequencies are 448 and 406 MHz, respectively, which are the resonance and off-resonance cases for the system at this displacement level. In Figure 2c,d, there is again a significant difference between the resonance and off-resonance cases in terms of localization. The magnitudes of the electric fields shown in these figures are of minor importance; the true value of this comparison is that it demonstrates we obtain a significantly high localization regardless of the displacement level when the NSRR is illuminated in the resonance of the coupled system corresponding to the current gap between the NSRR parts.

Higher localization of fields implies higher sensitivity since we can excite the NSRR effectively and the resonances become deeper due to the increase in stored energy in the near-field coupled system.

## 4. Experiments

Four different types of experimental sets are performed with the displacement sensor. As the first set of experiments, a controlled displacement is applied to separate the two parts of the NSRR by a translation stage in the laboratory-scale characterization setup that can move in 3-directions. The NSRR probe is fixed using sticky tape onto two pieces of cardboard individually screwed to the stage. When a displacement starts to occur on the structure on which the NSRR probe is placed, the local frequency peak of the coupled response of the sensing system is shifted. There is no hysteresis of the system; a certain frequency always corresponds to the same displacement level. In the second set of experiments, a parameter called tracking range is defined and its variation with the change of the distance between the antenna and the NSRR probe (Dm) is investigated. In the third set of experiments, the resolution of the sensor is examined by observing its response on μm-level displacements. The final experiments are performed on a large-scale mechanical loading setup, where a force is applied to a standard rebar in vertical direction, on which the NSRR probe is attached and the frequency shift in the elastic region of the rebar is monitored. The results are calibrated and then compared with the data from strain gages and extensometers which also simultaneously record data with our sensor. The details of the experiments and discussion about the results can be found in the next section.

## 5. Results and Discussion

**Figure 3.**
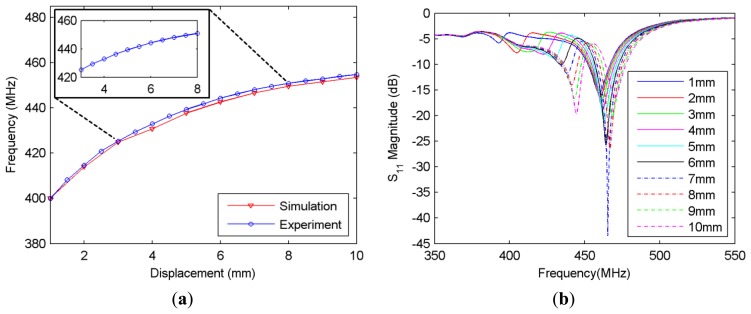
(**a**) The shift in the operating frequency measured as a function of the displacement d, presenting both simulation and experimental results; (**b**) Experimental S_11_ data that shows the shifting of the frequency peaks which are plotted in (a) for different displacement levels, d.

The first experiment set performed on the sensor is the translation stage based displacement characterization. The shift in frequency peaks *versus* the displacement is presented in Figure 3a for both the experimental and simulation results, while the shifting S_11_ magnitude curves are given in Figure 3b.

It can be observed in Figure 3a that the simulation results agree quite well with the experimental results. The simulation is again carried out in CST Microwave Studio. In the simulation, the jumper is modeled as a 4 cm-long solid wire with a radius of 0.1 mm. Two 3 mm-thick cardboard pieces (which can be seen behind the NSRR probe in Figure 1) are used to hold the two parts of the NSRR probe on the test setup and are also included in the simulation. As observed from the figure, the simulation and experimental results are substantially coherent. The agreement between the simulation and measurement results provides important validation of the simulation effort and establishes confidence in the predictions of the simulation for possible different scenarios.

In Figure 3a, the experimental data are the average of five different measurements. The antenna and the comb-like NSRR were separately designed and integrated to form the coupled response. A highly linear zoomed-in region of the frequency shift curve is also shown in the top left inset of Figure 3a. As a measure of linearity, *R*^2^ parameter is used.

As expected, for the sensor measurements, *R*^2^ starts to decrease as the displacement range is increased. The zoomed portion of the displacement range, which is 3–8 mm, has an *R*^2^ value of 0.990, indicating a very high linearity. The linear fit for a displacement range includes the system noise since the shifting peaks are detected from the measured data with an addition of environmental noise. Sensitivity can thus be defined as the slope of the frequency-displacement curve in Figure 3b (with a unit of MHz/mm). For various displacement ranges, *R*^2^ and sensitivity are listed in Table 1.

Although the linearity degrades as the displacement range is increased, it is still above 0.95 for a range of over 7 mm. If the maximum displacement range that yields an *R*^2^ value over 0.95 is defined as the dynamic range, then the dynamic range of the displacement sensor is found to be over 7 mm. As visible in Table 1, another advantage of the sensor is that it can be utilized in several displacement initial values without sacrificing its linear response. Thus, an initial separation can be allowed on the NSRR probe to create the frequency offset, and the sensor can be utilized in a higher-displacement region depending on the linearity and resolution of the region. As observed in Table 1, the *R*^2^ parameter is 0.99 for 3–8 mm. On the other hand, saturation is observed at high displacement levels, meaning that the rate of change of frequency with displacement decreases, and this leads to a lower sensitivity. Therefore, an optimum displacement range can be selected subject to the limitations set by the structure geometry and the desired operation frequency of the sensor. The maximum displacement attained by the sensor is more than enough for the purpose of SHM since normal elastic region strain levels that may be observed on the structural components are far less than what was measured. For instance, for a typical rebar, if we take the maximum elastic region strain level (yield strain) to be a few hundred microstrain, the relative displacement level corresponds to a few tens of μm (taking the starting length as the distance between the attachment points in the middle of the two comb-like NSRR parts and assuming a typical initial separation of 1 mm). On the other hand, determining the level of displacement experienced by a structural component after a big impact like an earthquake is also as critical as monitoring the typical service displacement levels. A measurement such as this can provide valuable quantitative information whether the evacuation of a damaged building will or will not be necessary. Therefore, the high dynamic range achieved by the displacement sensor makes it convenient for applications such as these. Given its dynamic range and sensitivity, the sensor can also be utilized in other applications where mm-scale displacement is to be monitored.

**Figure 4.**
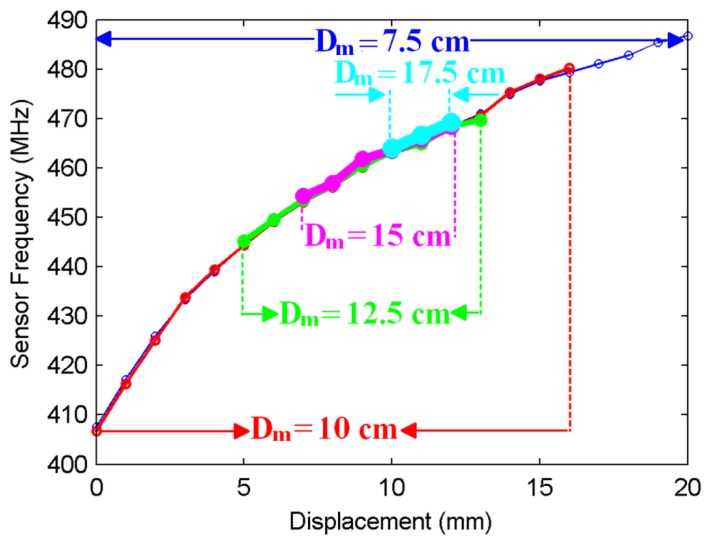
Displacement tracking range plotted for different monitoring distances *D_m_* (the distance between the antenna and the NSRR probe) at a tracking threshold of 1 dB. The tracking range diminishes as *D_m_* is increased.

In the second set of experiments, the displacement tracking range of the sensor is investigated. Displacement tracking range is defined as the maximum displacement range that can be captured by the antenna. This range can be expressed as the shift of the peaks that can be distinguished as a local maximum or minimum by a peak detection mechanism, e.g., a computer program. In order to determine the displacement tracking range, a threshold for tracking is desired. First, for the S_11_ magnitude curve (|S_11_| *versus* frequency curve like in Figure 3b) corresponding to the initial displacement level (*d* = *d_initial_*) which is the case where no force is applied, a local maximum or minimum is selected at or around the resonance frequency and defined as |S_11_|_initial_. Whenever a force is applied, a displacement *d* > *d_initial_* or *d* < *d_initial_* occurs leading to a new |S_11_| *versus* frequency curve. We record |S_11_| for each *d* (defined as |S_11_|_new_), and we monitor |S_11_|_new_ − |S_11_|_initial_. If, initially a local maximum point is chosen, the difference at this point will be in the form of a dip (if a local minimum is selected, it will be a peak). Then, tracking threshold corresponds to a preselected minimum difference in dB below which we do not consider the point as “tracked”. The tracking range is plotted for different monitoring distance levels in Figure 4 for a tracking threshold of 1 dB. It should be emphasized that the tracking range is closely related with the positions of the antenna and the NSRR with respect to each other. As the distance between the antenna and the NSRR probe (labeled as *D_m_*) is increased, this range is expected to decrease. On the other hand, the one-to-one relationship between a displacement level and the corresponding frequency is independent of the positions of the sensor elements. The positions only affect the tracking range; as long as the shift of a frequency peak is captured, it corresponds to the same displacement level, irrelevant of the antenna or NSRR probe position. As seen in the figure, when the distance between the antenna and the probe is 7.5 cm, the whole displacement range of 20 mm can be tracked by the sensor at the specified tracking threshold. However, the tracking range decreases as the antenna is moved away from the NSRR probe, and falls down to about 2 mm for a monitoring distance of 17.5 cm for the tracking threshold of 1 dB. As the tracking threshold is decreased, the tracking range naturally increases. However, reducing the tracking threshold too much complicates the detection, in case it becomes comparable with the system noise level.

**Figure 5.**
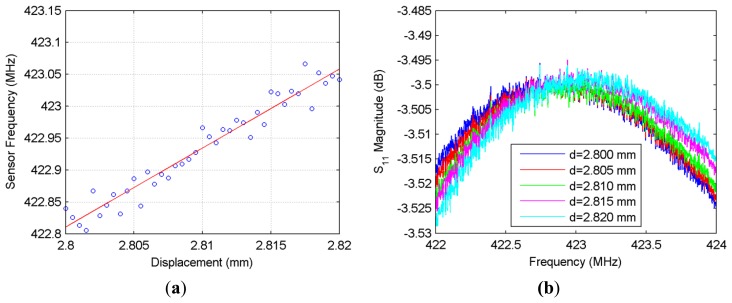
(**a**) Displacement sensor frequency shift *versus* a displacement range of 20 μm with 0.5 μm steps shown along with a linear fit; (**b**) S_11_ data that shows the shifting of the frequency peaks which are plotted in (a) for different displacement levels, d.

The resolution of the sensor is also a highly critical parameter. The third experiment demonstrates that the sensor is sensitive to submicron displacement levels. The frequency shifts for a range of 20 μm with 0.5 μm steps are plotted in Figure 5a. To monitor the frequency response of the coupled system, an Agilent FieldFox N9915A vector network analyzer was used. The graph that shows the S_11_ magnitude for different d values is given in Figure 5b.

The data displayed in Figure 5a represent the averaging option of the vector network analyzer, but no smoothing is applied in order to retain the system noise. In the experiments, IF bandwidth is set to be 10 kHz while the collected data are averaged over two measurement points. Fitting is applied to these curves in order to get rid of the effects of noise and then the local peaks of these fits are plotted. This figure reveals that the sensor is sensitive to a displacement as low as 0.5 μm. Taken together with our previous results, the sensor can detect displacements starting from the submicron level and extending up to 10 mm level. This high detection range allows the sensor to be used both in the elastic and the plastic deformation regions via displacement detection.

**Figure 6.**
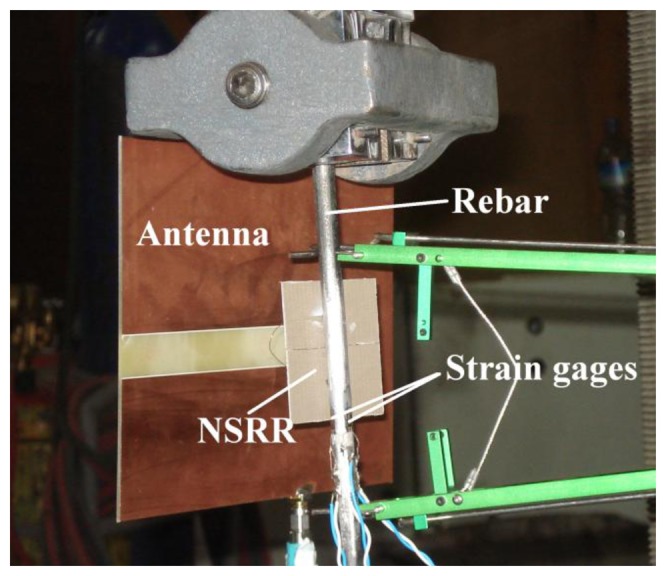
The rebar and sensor elements shown on the high-force mechanical setup.

As a final set of experiments, the sensor has also been tested on a standard rebar of 8-mm diameter with a high-force mechanical setup. In this setup, the rebar was pulled and elongated along a vertical direction in the elastic range, where the force was linearly increased from 0 up to 800 kgF. During this operation, the NSRR probe was fixed on the rebar in a position where the gap between the two parts of the probe could extend with the development of displacement along the vertical direction, as shown in Figure 6. Each part of the probe was fixed using strain gauge adhesive on the rebar in the form of a single 1-point attachment (over a small area of several mm's) for each part, to avoid the strain propagation through the hard epoxy onto the probe parts, which eliminates the probe detachment problem at high levels of strain. These point attachments were located at the central points of the two NSRR parts and the contact points were carefully pre-cleaned off rust. For the average strain calculations, the starting separation between the point attachments is thus taken as the half of the NSRR chip edge length, which is 2.35 cm.

**Figure 7.**
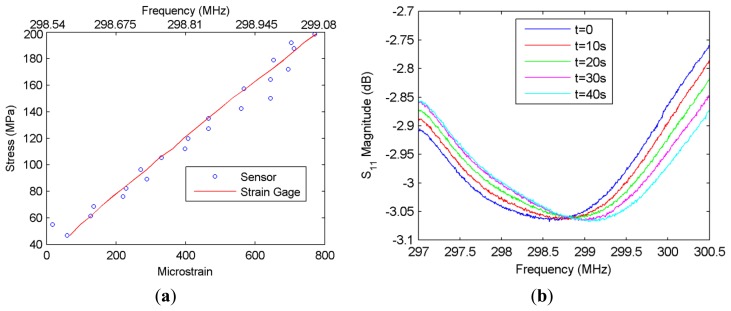
(**a**) Stress *versus* microstrain characteristics, experimentally measured by the sensor, in comparison to the average of the strain gage readings for an 8-mm diameter rebar pulled in a high-force mechanical setup. Corresponding resonance frequencies are also displayed on the upper horizontal-axis; (**b**) Shift of the frequency minima over time as the force is linearly increased, which is used to plot (a).

The antenna monitors the frequency shift shown in Figure 7b from a monitoring distance of approximately 12 cm, and the network analyzer records data at every 2 s. Simultaneously, three strain gages connected to the acquisition system with wires also collect data at exactly the same instants with the same time step of 2 s. The antenna and the strain gages can be seen in Figure 6. The positioning of the strain gages is such that one of them is located directly across the probe and the other two are placed at the two sides of the probe. The average of strains obtained from the gages placed at either side of the rebar is the axial strain. This is independent of the possible skewness and bending of the rebar which are usually observed due to the imperfections of the tensile test set-up. Thus, the average of strains obtained from the two strain gages facing each other are compared with the average strain calculated from the sensor reading and the strain gage across the sensor. A calibration is needed to convert the frequency shift information of the sensor into the strain. For this purpose, the data obtained from the first experiment are utilized. The average strain values obtained from the gages at each time instant are used for the transformation of the frequency shift into the strain in a linear fashion, and this transformation acts as a calibration for the other measurements. Then, the stress calculated from the applied force (by dividing the force by the initial rebar area) can be presented as a function of the microstrain forming in the vertical direction on the rebar, as shown in Figure 7a. For the elastic region, this curve is expected to be linear, and in fact this is what is observed in the plot from the strain gage measurements (Figure 7a). The sensor readings are also observed to be closely following this line, demonstrating that the displacement sensor can also function efficiently in the presence of a rebar touching the probe under high axial forces.

It should be emphasized that in this particular measurement, the frequency shift is directly converted into strain instead of displacement, since it is compared with the information obtained from the strain gages. It should also be noted that strain gages measure strains at the points they are fixed whereas strain calculated from the sensor is actually the average of strain occurring within the height of the sensor. Thus, it was assumed that these two strain levels are comparable since the rebar is under constant axial stress. With this assumption, the displacement and strain information can both be acquired by the sensor and they are convertible to each other as long as we know the initial separation between the two parts of the NSRR probe.

An important point about the high-scale loading experiments is that the measurements are performed in free space, *i.e.*, there is no object (*i.e.*, no scattering or absorbing medium) present between the antenna and the NSRR probe. However, in a real-life scenario, generally the rebar is embedded into concrete. The combination of rebar and concrete will form an electromagnetically complex medium, which may generate a disruptive effect on microwave signals in certain bands. It is clear that the resulting complex medium will thus affect the coupled sensor system. Our high-force mechanical setup experiments here demonstrate that the resonance frequencies drop down by about 100 MHz compared to the laboratory-scale displacement characterization (based on translation stage measurements without a rebar) with the presence of an 8-mm diameter rebar behind the NSRR (see Figure 6a, upper horizontal axis). In the high-level loading experiments using a rebar, it is also shown that the resonance frequency shift is still observed despite the rebar and the sensor is capable of capturing it in this case. The probable effect of the rebar-concrete composite on the sensor is to increase the attenuation and scattering of the microwave signals, therefore making it more difficult to detect the frequency peaks.

## 6. Conclusions/Outlook

In conclusion, this work proposes and demonstrates a displacement sensor that can be utilized to detect μm-scale to mm-scale displacements experienced in a structure. The sensor is primarily designed for use in SHM and post-earthquake damage assessment. The sensor exhibits a high linearity (where *R*^2^ reaches 0.99 for a range of 5 mm) and a high resolution (monitoring displacement changes as low as <1 μm). The dynamic range of the sensor is as high as 7 mm when it is defined as the range where *R*^2^ > 0.95. The sensor is also shown to be operating seamlessly in the linear region as attached on a rebar. A primary advantage of the sensor is that it is not limited by the Young's modulus of the material of the NSRR probe because the separation of the comb-like NSRR geometry into two electrically shorted parts allows the measurement of high displacement levels. As shown in a proof-of-concept demonstration here, since the comb-like NSRR is passive and the sensor can track displacement remotely, the design eliminates the complications due to destructive testing and requirement of replacing the power source. As future steps, the effect of concrete on the sensor performance is going to be investigated to test the real-life scenario performance of the sensor. Also, experiments using rebars embedded into concrete will be undertaken. As a proof-of-concept demonstration, the experimental findings presented here, also supported by numerical modeling, suggest that displacement sensors enabled by such metamaterial probes hold great promise to be utilized in rebar-based structural components for remote SHM.

## Figures and Tables

**Table 1.** Linearity and sensitivity of the displacement sensor: *R*^2^ and sensitivity parameters for several selected displacement ranges.

**Displacement range (*d****_min_****−d****_max_***) (mm)**	***R*^2^**	**Sensitivity (MHz/mm)**
1–3	0.988	12.70
1–6	0.970	8.86
1–8	0.955	6.61
2–7	0.984	6.69
3–8	0.990	5.12
3–7	0.995	5.68
4–8	0.991	4.48
